# Whole-genome sequencing to explore nosocomial transmission and virulence in neonatal methicillin-susceptible *Staphylococcus aureus* bacteremia

**DOI:** 10.1186/s13756-020-0699-8

**Published:** 2020-02-22

**Authors:** Bibi C. G. C. Slingerland, Margreet C. Vos, Willeke Bras, René F. Kornelisse, Dieter De Coninck, Alex van Belkum, Irwin K. M. Reiss, Wil H. F. Goessens, Corné H. W. Klaassen, Nelianne J. Verkaik

**Affiliations:** 1 Department of Medical Microbiology and Infectious Diseases, Erasmus MC, University Medical Center Rotterdam, Dr. Molewaterplein 40, 3015 GD Rotterdam, The Netherlands; 2 Department of Pediatrics, Division of Neonatology, Erasmus MC, University Medical Center Rotterdam, Rotterdam, The Netherlands; 3 BioMérieux SA, Data Analytics, Clinical Unit, Sint-Martens-Latem, Belgium; 4 BioMérieux SA, Clinical Unit, 38390 La Balme-les-Grottes, France

**Keywords:** *Staphylococcus aureus*, Bacteremia, Whole-genome sequencing, Neonatal intensive care unit, Transmission

## Abstract

**Background:**

Neonatal *Staphylococcus aureus* (*S. aureus*) bacteremia is an important cause of morbidity and mortality. In this study, we examined whether methicillin-susceptible *S. aureus* (MSSA) transmission and genetic makeup contribute to the occurrence of neonatal *S. aureus* bacteremia.

**Methods:**

A retrospective, single-centre study was performed. All patients were included who suffered from *S. aureus* bacteremia in the neonatal intensive care unit (NICU), Erasmus MC-Sophia, Rotterdam, the Netherlands, between January 2011 and November 2017. Whole-genome sequencing (WGS) was used to characterize the *S. aureus* isolates, as was also done in comparison to reference genomes. Transmission was considered likely in case of genetically indistinguishable *S. aureus* isolates.

**Results:**

Excluding coagulase-negative staphylococci (CoNS), *S. aureus* was the most common cause of neonatal bacteremia. Twelve percent (*n* = 112) of all 926 positive blood cultures from neonates grew *S. aureus*. Based on core genome multilocus sequence typing (cgMLST), 12 clusters of genetically indistinguishable MSSA isolates were found, containing 33 isolates in total (2–4 isolates per cluster). In seven of these clusters, at least two of the identified MSSA isolates were collected within a time period of one month. Six virulence genes were present in 98–100% of all MSSA isolates. In comparison to *S. aureus* reference genomes, toxin genes encoding staphylococcal enterotoxin A (*sea*) and toxic shock syndrome toxin 1 (*tsst-1*) were present more often in the genomes of bacteremia isolates.

**Conclusion:**

Transmission of MSSA is a contributing factor to the occurrence of *S. aureus* bacteremia in neonates. *Sea* and *tsst-1* might play a role in neonatal *S. aureus* bacteremia.

## Introduction

*Staphylococcus aureus* (*S. aureus*) is a well-established nosocomial pathogen that causes multiple types of neonatal infections [1, 2]. Invasive *S. aureus* infections in neonates (e.g. bacteremia) are common in very low birth weight (VLBW) infants, which makes this bacterial species one of the most important pathogens in neonatal intensive care units (NICU) [3–5]. A significant risk factor for *S. aureus* bacteremia in VLBW infants is the presence of intravascular catheters, which are frequently required [6–8]. In addition, *S. aureus* bacteremia can result in severe complications such as endocarditis and osteomyelitis [5, 9, 10]. All-cause mortality among neonates suffering from *S. aureus* bacteremia varies between 10 and 20% [7, 11]. So there is an urgent need to prevent this infection. To prevent *S. aureus* bacteremia in neonates, it is important to know the factors contributing to the high frequency and severity of this infection.

Previously, the virulence factors *tsst-1* and *sea* were implicated to play a role in *S. aureus* bacteremia [12–14]. Furthermore, transmission of *S. aureus* might contribute to the high frequency of bacteremia. Outbreaks of methicillin-resistant *S. aureus* (MRSA) at the NICU are described and relatively easy to detect [15–18]. Meanwhile, the detection of methicillin-sensitive *S. aureus* (MSSA) outbreaks seems to be more difficult, excluding outbreaks in patients who suffer from a skin infection [19–22]. In this study, whole-genome sequencing (WGS), the typing method with the highest discriminatory power, was used to determine whether MSSA transmission and genetic makeup, contribute to the occurrence of neonatal *S. aureus* bacteremia.

## Methods

### Population

The NICU of Erasmus MC-Sophia, Rotterdam, the Netherlands, is a level IV, 27-beds facility. It is divided into four units with six to eight beds each. Per year, about 750 neonates are admitted. Nearly 40% of them are below 32 weeks of gestation and were in majority born in this hospital.

### Screening

We included neonates with a presumed infection, of whom blood cultures were obtained between January 2011 and November 2017 that showed to be positive for *S. aureus*. Clinical data concerning gender, gestational age, birth weight and survival were obtained from patient records.

### *S. aureus* isolates

Blood from neonates was cultured in BACTEC plus PEDS aerobic bottles and incubated in the Bactec FX (BD, Heidelberg, Germany). In case of positive blood cultures, plates were inoculated and, after 16–24 h of incubation at 37 °C, screened for *S. aureus* based on colony morphology. Identification was performed by means of a latex agglutination test (Slidex Staph Plus, bioMérieux, Marcy-l’Etoile, France) and/or via matrix-assisted laser desorption/ionisation, time-of-flight, mass spectrometry (MALDI-TOF MS system, Bruker). *S. aureus* isolates were stored at − 20 °C or – 80 °C until use. The VITEK 2 system (bioMérieux) was used for antimicrobial susceptibility testing (AST).

### Whole-genome sequencing

#### Transmission

*S. aureus* isolates were processed according to the bioMérieux EpiSeq^cs^ V1 programme and sent to LGC Genomics GmbH (Berlin, Germany) for next-generation sequencing (NGS). We used Illumina chemistry, which generated paired end 2 × 150 bp reads. Sequences were assembled using the proprietary built-in assembler from CLC Genomics Workbench v11 software (Qiagen, Hilden, Germany) with default parameters. We analysed them by means of the available *S. aureus* core genome multilocus sequence typing scheme (cgMLST) [23] in BioNumerics 7.6.3 (bioMérieux, Sint-Martens-Latem, Belgium) which contains 1861 loci. Allele calling was performed using two algorithms, one based on the assembly using a BLAST approach (assembly-based calling) and one based on the trimmed sequencing data using a kmer based approach (assembly-free calling). A consensus of both algorithms was used to assign final allele calls: when both algorithms were in agreement or when an allele call was made by only one of the algorithms, the allele call was considered in the consensus. However, when both algorithms were in disagreement, the allele call was not considered in the consensus. Both allele calling algorithms were executed using default parameters. Conventional MLST types were inferred in silico from the WGS data. To this end, the seven MLST loci were identified using the sequence extraction tool and the MLST plugin from BioNumerics 7.6.3 that is synchronized to the pubMLST.org public repository (accession date: April 5, 2019). For the visualisation of the genetic relatedness between the isolates, we used a minimum spanning tree for the cgMLST data. The MST was generated using default parameters, and no re-sampling was performed. Isolates containing less than 12 allelic differences in the *S. aureus* core genome were considered genetically indistinguishable [23]. We defined a cluster as more than two genetically indistinguishable isolates and, within a cluster, considered transmission of *S. aureus* likely. To further validate the results based on the cgMLST approach, as additional method, we evaluated transmission events using a SNP based approach (Additional file 1: Table S1).

#### Virulence

The presence of virulence genes was assessed, using the sequence extraction tool in BioNumerics 7.6.3. Extraction parameters (percentage coverage and identity) were individualised to accommodate for the different levels of sequence diversity within and between the virulence genes. Anticipating problems upon assembling virulence genes containing repetitive motifs (*sdrA*, −*B* and -*C*, *clfA* and –*B*, *cna*, *sasG*) using the short read sequence data, only the largest non-repetitive part of these genes was used for quering. In order to obtain data from a general *S. aureus* population, the prevalence of virulence genes was also assessed by means of the available genomic sequences in the Refseq Genome Database, using the BLAST interface (https://blast.ncbi.nlm.nih.gov/Blast.cgi). This database contained 10,288 *S. aureus* genomes at the time of analysis. Virulence gene-specific search parameters were used as discussed above. Role and function of the *S. aureus* virulence genes were described in more detail earlier [12, 24]. An overview of analysed virulence genes, their role, search parameters and query sequence are shown in Additional file 2: Table S2.

## Results

### Patient characteristics

After coagulase-negative staphylococci (CoNS), MSSA was the most frequent causative pathogen of bacteremia in neonates. Several species of CoNS were isolated from neonatal blood, but they were considered to be one group. Twelve percent (*n* = 112) of 926 positive blood cultures from neonates (one blood culture per episode per patient), taken in the period January 2011 – November 2017, were positive for MSSA. Fifty-nine of the 112 neonates (52.7%) with MSSA bacteremia were male. The median (interquartile range) for gestational age and birth weight were 26 3/7 (25 1/7–30) weeks and 880 (680–1150) grams, respectively. The onset of all episodes of MSSA bacteremia occurred 72 h after birth, at a median postnatal age of 10 (7–19) days. The overall mortality among the included 112 patients was 20.5% while 11 of these 23 neonates died of MSSA septicaemia.

### Genetic relatedness

**Fig. 1:**
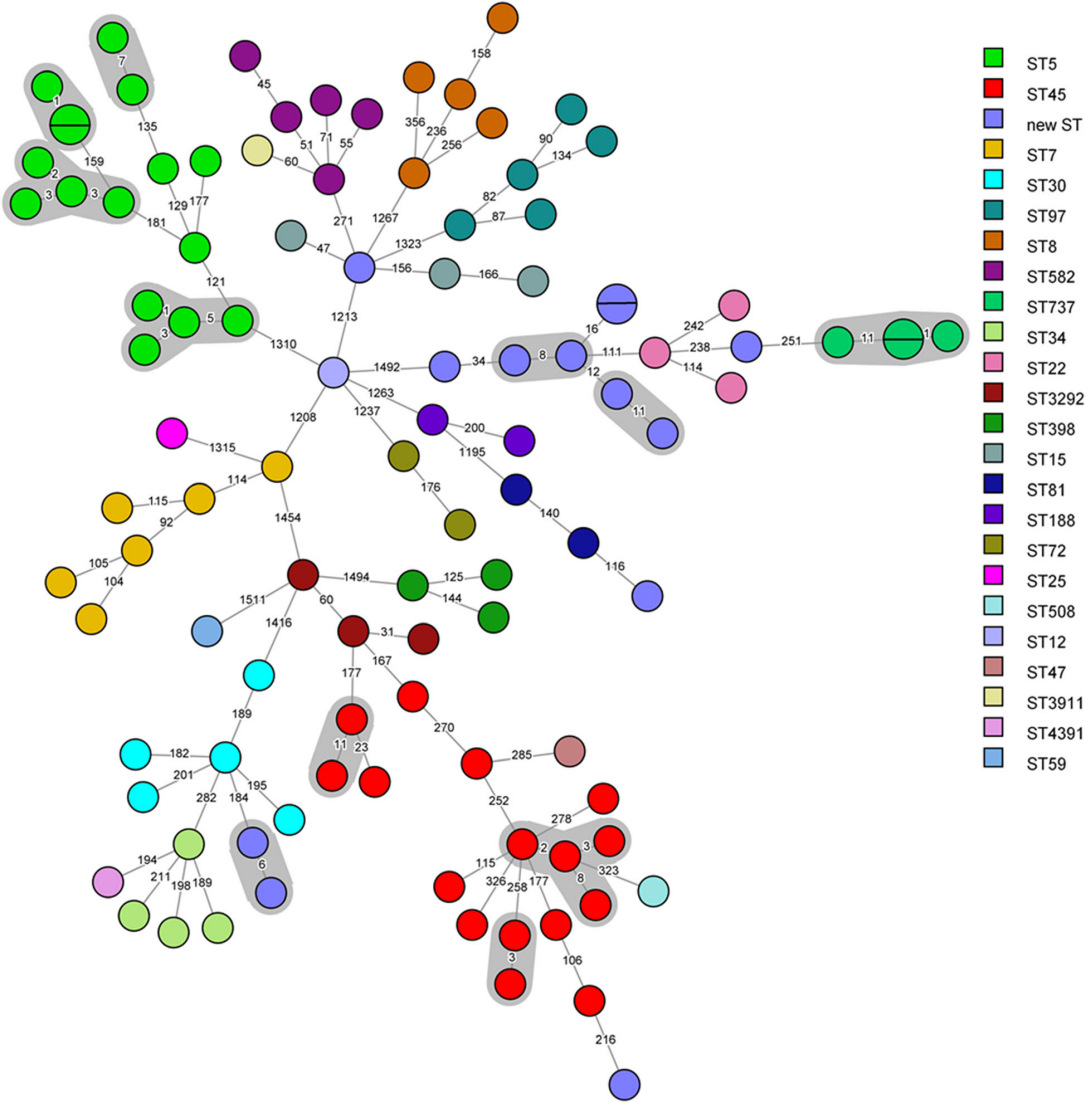
Minimum spanning tree, based on the core genome of 104 *S. aureus* isolates. Colours indicate the classical MLST sequence types (ST). Twelve cgMLST clusters containing at least two isolates with a maximum of eleven allelic differences are indicated with a grey background

One hundred and four MSSA isolates from the total of 112 neonatal bloodstream isolates (93%) were available and therefore included for WGS (including only the first isolate per patient). Based on WGS, a total of 23 classical MLST types were identified. The most predominant MLST types were ST5 and ST45 (for both *n* = 16). For 11 MSSA isolates a novel MLST type was found. To assess the genetic relatedness between the 104 isolates based on the more discriminatory cgMLST scheme, we visualised the number of allelic differences of the isolates in Fig. 1. Twelve cgMLST clusters of genetically indistinguishable isolates were observed, containing a total of 33 isolates (2–4 isolates per cluster). In seven of these cgMLST clusters, at least two of the identified MSSA isolates were collected within a time period of one month. In two cgMLST clusters, all MSSA isolates were found within a time period of one year, but the shortest time interval between isolates of two neonates was forty days. In the other three cgMLST clusters, there was a time interval of more than one year between culturing the MSSA bloodstream isolates of two neonates. The SNP approach confirmed our results based on the cgMLST approach (Additional file 1: Table S1).

### *S. aureus* virulence genes

An overview of virulence genes present in the 104 MSSA isolates is provided in Table 1. Of the immunomodulatory proteins, staphylococcal complement inhibitor (*scin*) was present in 100% of all bloodstream isolates. Alpha-hemolysin (*hla*) was present in 99% of the isolates. We also found a 98–100% presence of the MSCRAMMs clumping factors A and B (*clfA*, *clfB*), immunodominant surface antigen A (*isaA*) and iron-responsive surface determinants A and H (*isdA*, *isdH*). When compared to a reference population of *S. aureus* genomes, a few observations stand out. Remarkably, staphylococcal enterotoxin A (*sea*) and toxic shock syndrome toxin 1 (*tsst-1*) were, respectively, 2.6 and 3.2 times more prevalent among the 104 neonatal bloodstream isolates, relative to the reference genomes. Likewise, staphylococcal enterotoxin h (*seh*) was 3.4 times more prevalent although, in absolute numbers, this involved only a few isolates (6/104 versus 173/10288). For the other virulence genes, no such increases were detected (Table 1).

**Table 1:** Presence of virulence genes in neonatal *S. aureus* isolates compared to reference genomes

genes	neonatal isolates (%)	refseq (%)	genes	neonatal isolates (%)	refseq (%)
*sea*	24.0	9.4	*cna*	51.0	34.6
*seb*	4.8	5.9	*eap-map*	64.4	93.7
*sec*	18.3	10.8	*ebp*	95.2	95.2
*sed*	10.6	8.8	*fnbpA*	66.3	75.2
*seg*	51.9	55.3	*fnbpB*	65.4	75.0
*seh*	5.8	1.7	*sdrC*	91.3	96.3
*sei*	55.8	55.2	*sdrD*	50.0	74.9
*sej*	10.6	11.1	*sdrE*	89.4	85.0
*sek*	3.8	19.0	*efb*	77.9	94.7
*sel*	18.3	10.8	*icaA*	100.0	97.8
*sem*	55.8	55.6	*icaB*	89.4	98.5
*sen*	45.2	55.2	*icaC*	99.0	97.8
*seo*	58.7	49.1	*icaD*	100.0	98.0
*sep*	4.8	19.1	*icaR*	95.2	97.7
*seq*	4.8	19.1	*isaA*	100.0	98.3
*ser*	10.6	10.9	*isdA*	100.0	97.6
*ses*	0.0	0.3	*isdH*	100.0	97.5
*set*	0.0	0.3	*sasG*	54.8	56.7
*seu*	51.9	54.8	*edn*	1.0	1.4
*sey*	1.0	5.8	*etA*	1.0	1.0
*aur*	100.0	98.2	*etB*	0.0	0.2
*coa*	70.2	83.0	*etD*	1.0	0.8
*geh*	87.5	97.5	*hla*	99.0	97.1
*hysA*	98.1	96.5	*hlb*	100.0	95.1
*sak*	73.1	78.5	*hld*	90.4	98.0
*sspA*	99.0	98.1	*hlgA*	96.2	97.6
*sspB*	91.3	98.1	*hlgB*	100.0	97.8
*sspC*	100.0	98.3	*hlgC*	98.1	97.5
*vWbp*	78.8	98.1	*lukD*	49.0	66.5
*adsA*	96.2	98.1	*lukE*	49.0	65.9
*chp*	68.3	67.2	*lukF*	0.0	18.8
*sbi*	100.0	97.7	*lukM*	0.0	0.7
*scn*	98.1	98.2	*lukS*	0.0	19.0
*clfA*	98.1	97.4	*tsst-1*	18.3	5.8
*clfB*	100.0	97.8			

## Discussion

At our level IV neonatal intensive care unit, as in many centres [3–5], *S. aureus* is a frequent cause of neonatal bacteremia. In our study, we explored the role of MSSA transmission and the possible contribution of virulence genes. By using WGS, 12 different cgMLST clusters of MSSA isolates were found. Seven of these twelve cgMLST clusters included at least two MSSA isolates, cultured from blood of neonates within one month, indicative for transmission. Transmission should therefore be considered as a contributing factor for the frequent occurrence of neonatal *S. aureus* bacteremia, as was recently described by *Rouard* et al. [13]. Although it seems reasonable to assume that transmission, irrespective of the source, can only occur through the hands of healthcare workers (HCWs), we did not prove this, since we did not culture the environment, nor the HCWs or parents. Still, general measures such as improvement of the current (daily) cleaning, disinfection procedures as well as hand hygiene, will be likely to help. It was already proven that neonatal hospital-acquired infections could in part be prevented by strict infection control measures [8, 25, 26]. In addition, reinforcement of the implementation of central-line bundles has the potential to reduce the incidence of central line-associated bloodstream infections (CLABSIs); although these bundles are already implemented, compliance can still be improved and additional measures can be explored [27].

Besides transmission, it was determined whether the presence of certain virulence factors is associated with neonatal *S. aureus* bacteremia. Since it was difficult to define a suitable control population of neonates, we chose to compare neonatal *S. aureus* bacteremia isolates to all available *S. aureus* genomes from the Refseq Genome Database (*N* = 10,288 at the time of analysis). Remarkably, the genes *sea* and *tsst-1* were found a factor 2.6 and 3.2 times more often in the MSSA bloodstream isolates, compared to the reference genomes in the Refseq Genome Database. The overrepresentation of *tsst-1* could not be explained by the frequent presence of MLST ST5 and ST45 in our isolates collection, since *tsst-1* was not associated with these sequence types. On the other hand, 11 of the 25 isolates carrying *sea* were found in ST5 isolates. Still, this cannot be the full explanation for finding an association between *sea* and neonatal MSSA bacteremia. Many studies have been executed on *S. aureus* toxins and their pathogenic roles, particularly on *sea* and *tsst-1*. Previously, it was described that antibody responses to these two specific toxins were higher in patients with *S. aureus* bacteremia, compared to control patients [12]. In addition, in a recent publication about a NICU MSSA outbreak, *tsst-1* and especially *sea* were found in bloodstream isolates, compared to colonisation isolates [13]. Another review article describes the association of these toxins with bacteremia [14]. Therefore, this may suggest that *sea* and *tsst-1* might play a role in the pathogenesis of *S. aureus* bacteremia. The other virulence genes were present in virtually all study isolates, but in virtually all reference genomes as well (Table 1).

Our study has its limitations. It was performed retrospectively, in a single centre. We considered less than 12 allelic differences in the *S. aureus* core genome as indistinguishable, as described by *Leopold* et al. for MRSA outbreaks [23]. Still, it is a matter of debate which cut-off should be used to define MSSA isolates as indistinguishable. If the cut-off would have been set at 20 alleles [28], this would have led to 10 larger instead of 12 indistinguishable MSSA clusters, which would not have changed the conclusion regarding transmission. Additional studies are needed to define a clear cut-off. Finally, we compared neonatal isolates to a large number of reference genomes, but these originate from several countries, several clinical sites and patients of all ages. It would have been ideal if the reference genomes had originated from colonized but not infected neonates, admitted to the same NICU, in the same time period.

## Conclusions

In conclusion, transmission of MSSA seems a contributing factor to the occurrence of *S. aureus* bacteremia in neonates. The possibility of MSSA transmission in neonatal intensive care should be explored to prevent this invasive and serious infection. The exact role of *sea* and *tsst-1* warrants further investigation.

## Supplementary information

**Additional file 1:** **Table S1** Whole-genome Single-Nucleotide Polymorphism analysis

**Additional file 2:** **Table S2** *S. aureus* virulence genes

## Data Availability

The data generated and analysed in this study are included in the current article.
